# Tris(3-amino­phen­yl)phosphine oxide ethanol solvate

**DOI:** 10.1107/S160053680900909X

**Published:** 2009-03-25

**Authors:** Jun Han, Wenguang Li, Shufang Wang, Juli Jiang

**Affiliations:** a Key Laboratory of Mesoscopic Chemistry of the Ministry of Education, and School of Chemistry and Chemical Engineering, Nanjing University, Nanjing 210093, People’s Republic of China; b Jiangguantun Middle School, Liaocheng 252022, Shangdong Province, People’s Republic of China

## Abstract

The title compound crystallized as an ethanol solvate, C_18_H_18_N_3_OP·C_2_H_6_O. It is the reduction product of tris­(3-nitro­phen­yl)phosphine oxide. In the crystal, there are inter­molecular N—H⋯O hydrogen bonds between neighbouring tris­(3-amino­phen­yl)phosphine oxide mol­ecules and O—H⋯O hydrogen bonds involving the ethanol solvent mol­ecule.

## Related literature

The structure of tris­(3-nitro­phen­yl)phosphine oxide is described by Jean-Noël *et al.* (2004 ▶). For literature on related compounds, see: Michaelis *et al.* (1885 ▶); Dressick *et al.* (2000 ▶); Hessler & Stelzer (1997 ▶).
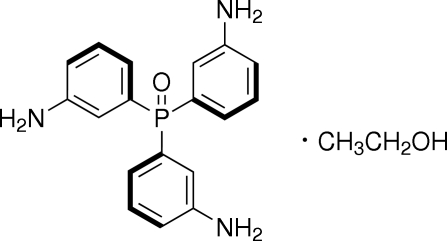


## Experimental

### 

#### Crystal data

C_18_H_18_N_3_OP·C_2_H_6_O
*M* *_r_* = 369.39
Triclinic, 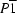

*a* = 9.1046 (13) Å
*b* = 10.7595 (15) Å
*c* = 12.020 (3) Å
α = 109.131 (3)°
β = 94.245 (3)°
γ = 114.028 (2)°
*V* = 986.3 (3) Å^3^
*Z* = 2
Mo *K*α radiation
μ = 0.16 mm^−1^
*T* = 293 K
0.35 × 0.34 × 0.30 mm

#### Data collection

Bruker SMART CCD area-detector diffractometer
Absorption correction: multi-scan (*SADABS*; Bruker, 2005 ▶) *T*_min_ = 0.947, *T*_max_ = 0.954
5014 measured reflections
3420 independent reflections
1659 reflections with *I* > 2σ(*I*)
*R*_int_ = 0.058

#### Refinement

*R*[*F* ^2^ > 2σ(*F* ^2^)] = 0.059
*wR*(*F* ^2^) = 0.149
*S* = 0.85
3420 reflections
174 parameters
H-atom parameters constrained
Δρ_max_ = 0.53 e Å^−3^
Δρ_min_ = −0.41 e Å^−3^

### 

Data collection: *SMART* (Bruker, 2005 ▶); cell refinement: *SAINT* (Bruker, 2005 ▶); data reduction: *SAINT*; program(s) used to solve structure: *SHELXS97* (Sheldrick, 2008 ▶); program(s) used to refine structure: *SHELXL97* (Sheldrick, 2008 ▶); molecular graphics: *SHELXTL* (Sheldrick, 2008 ▶); software used to prepare material for publication: *SHELXTL*.

## Supplementary Material

Crystal structure: contains datablocks global, I. DOI: 10.1107/S160053680900909X/pk2158sup1.cif

Structure factors: contains datablocks I. DOI: 10.1107/S160053680900909X/pk2158Isup2.hkl

Additional supplementary materials:  crystallographic information; 3D view; checkCIF report

## Figures and Tables

**Table 1:** Hydrogen-bond geometry (Å, °)

*D*—H⋯*A*	*D*—H	H⋯*A*	*D*⋯*A*	*D*—H⋯*A*
N3—H3*A*⋯N1^i^	0.86	2.62	3.469 (6)	168
N2—H2*B*⋯O1^ii^	0.86	2.14	2.987 (4)	168
N2—H2*C*⋯O2^iii^	0.86	2.23	3.089 (5)	173
O2—H2⋯O1	0.82	1.85	2.672 (3)	178

Symmetry codes: (i) 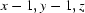; (ii) 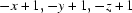; (iii) 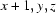.

**Fig. 1.**
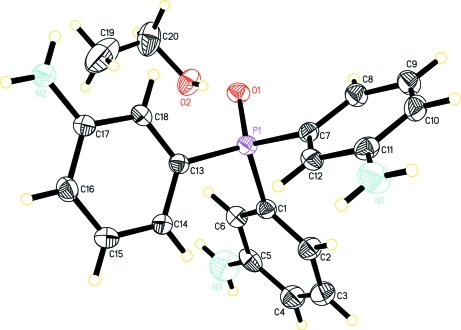
The molecular structure of the title compound with the atom-numbering scheme.

**Fig. 2.**
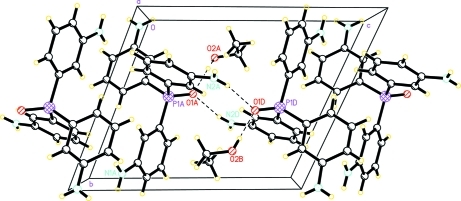
The crystal packing of the title compound, viewed along the a axis.

## supplementary crystallographic information

### Comment

Arylphosphines have been investigated extensively as ionic ligands for catalytically active transition metals in aqueous solution (Hessler & Stelzer, 1997), as starting materials for the molecular fabrication of materials (Dressick *et al.*, 2000) and so on. As early as 1885, tris(3-aminophenyl)phosphine oxide had been synthesized in the Sn/HCl system but with low yield (Michaelis *et al.*, 1885). The molecules of the title compound crystallized as an ethanol solvate (Fig. 1). Adjacent molecules are linked *via* intermolecular O—H···O and N—H···O interactions, such as O2—H2···O1, N2—H2B···O1, N2—H2C···O2 and N1—H1A···O2 from a neighboring molecule (Fig. 2).

### Experimental

The precursor, tris(3-nitrophenyl)phosphine oxide (1.032 g, 2.5 mmol), was added to a mixture of ethanol (30 ml), THF (30 ml), hydrazine hydrate (10 ml) and a catalytic amount of Raney Ni in a 100 ml flask. The mixture was heated to reflux and reaction progress was monitored by TLC.  The pure product was obtained as colorless crystals suitable for X-ray analysis after removing most of the solvent and without further purification (yield > 99%).

### Refinement

All the H atoms were positioned geometrically and refined using a riding model, with C—H = 0.93–0.97 Å and with *U*_iso_(H) = 1.2*U*_eq_(C), (1.5*U*_eq_(C) for methyl groups), and with a distance of O—H = 0.82 Å and *U*_iso_(H) = 1.5*U*_eq_(O), and N—H = 0.86 Å with *U*_iso_(H) = 1.2*U*_eq_(N). Although the diffraction data were rather weak, the structure is unambiguous, nevertheless, the ethanol solvent molecule is rather poorly defined.

### Crystal data

**Table:**

C_18_H_18_N_3_OP·C_2_H_6_O	*Z* = 2
*M**_r_* = 369.39	*F*(000) = 392
Triclinic, *P*1	*D*_x_ = 1.244 Mg m^−^^3^
Hall symbol: -P 1	Mo *K*α radiation, λ = 0.71073 Å
*a* = 9.1046 (13) Å	Cell parameters from 706 reflections
*b* = 10.7595 (15) Å	θ = 2.6–19.5°
*c* = 12.020 (3) Å	µ = 0.16 mm^−^^1^
α = 109.131 (3)°	*T* = 293 K
β = 94.245 (3)°	Prism, colorless
γ = 114.028 (2)°	0.35 × 0.34 × 0.30 mm
*V* = 986.3 (3) Å^3^	

### Data collection

**Table:**

Bruker SMART CCD area-detector diffractometer	3420 independent reflections
Radiation source: fine-focus sealed tube	1659 reflections with *I* > 2σ(*I*)
graphite	*R*_int_ = 0.058
φ and ω scans	θ_max_ = 25.0°, θ_min_ = 2.3°
Absorption correction: multi-scan (*SADABS*; Bruker, 2005)	*h* = −10→10
*T*_min_ = 0.947, *T*_max_ = 0.954	*k* = −11→12
5014 measured reflections	*l* = −14→14

### Refinement

**Table:**

Refinement on *F*^2^	Primary atom site location: structure-invariant direct methods
Least-squares matrix: full	Secondary atom site location: difference Fourier map
*R*[*F*^2^ > 2σ(*F*^2^)] = 0.059	Hydrogen site location: inferred from neighbouring sites
*wR*(*F*^2^) = 0.149	H-atom parameters constrained
*S* = 0.85	*w* = 1/[σ^2^(*F*_o_^2^) + (0.0599*P*)^2^] where *P* = (*F*_o_^2^ + 2*F*_c_^2^)/3
3420 reflections	(Δ/σ)_max_ < 0.001
174 parameters	Δρ_max_ = 0.53 e Å^−^^3^
0 restraints	Δρ_min_ = −0.41 e Å^−^^3^

### Special details

**Table:**

Geometry. All e.s.d.'s (except the e.s.d. in the dihedral angle between two l.s. planes) are estimated using the full covariance matrix. The cell e.s.d.'s are taken into account individually in the estimation of e.s.d.'s in distances, angles and torsion angles; correlations between e.s.d.'s in cell parameters are only used when they are defined by crystal symmetry. An approximate (isotropic) treatment of cell e.s.d.'s is used for estimating e.s.d.'s involving l.s. planes.
Refinement. Refinement of *F*^2^ against ALL reflections. The weighted *R*-factor *wR* and goodness of fit *S* are based on *F*^2^, conventional *R*-factors *R* are based on *F*, with *F* set to zero for negative *F*^2^. The threshold expression of *F*^2^ > 2σ(*F*^2^) is used only for calculating *R*-factors(gt) *etc*. and is not relevant to the choice of reflections for refinement. *R*-factors based on *F*^2^ are statistically about twice as large as those based on *F*, and *R*- factors based on ALL data will be even larger.

### Fractional atomic coordinates and isotropic or equivalent isotropic displacement parameters (Å^2^)

**Table:**

	*x*	*y*	*z*	*U*_iso_*/*U*_eq_	
P1	0.32847 (11)	0.46681 (10)	0.24048 (9)	0.040	
O1	0.2533 (3)	0.4519 (2)	0.3452 (2)	0.048	
O2	0.0137 (3)	0.2243 (3)	0.3724 (3)	0.0692 (9)	
H2	0.0879	0.2929	0.3632	0.104*	
C12	0.5751 (4)	0.7116 (4)	0.2172 (3)	0.0450 (9)	
H12	0.6148	0.6475	0.1749	0.054*	
C7	0.4373 (4)	0.6580 (4)	0.2618 (3)	0.0398 (9)	
C1	0.1745 (4)	0.3739 (4)	0.0993 (3)	0.0430 (9)	
C13	0.4720 (4)	0.3909 (3)	0.2210 (3)	0.0380 (9)	
C17	0.6849 (4)	0.3539 (4)	0.3165 (3)	0.046	
C11	0.6559 (4)	0.8607 (4)	0.2346 (3)	0.049	
C18	0.5679 (4)	0.4064 (3)	0.3238 (3)	0.044	
H18	0.5539	0.4528	0.3996	0.052*	
N1	0.7909 (4)	0.9160 (4)	0.1905 (3)	0.076	
H1A	0.8373	1.0081	0.2021	0.091*	
H1B	0.8298	0.8588	0.1510	0.091*	
C14	0.4895 (4)	0.3209 (4)	0.1081 (3)	0.048	
H14	0.4243	0.3095	0.0387	0.058*	
C16	0.7007 (4)	0.2837 (4)	0.2010 (3)	0.052	
H16	0.7776	0.2472	0.1931	0.062*	
C6	0.0464 (4)	0.2340 (4)	0.0766 (3)	0.0486 (10)	
H6	0.0446	0.1908	0.1324	0.058*	
C5	−0.0779 (4)	0.1596 (4)	−0.0291 (4)	0.057	
C2	0.1779 (5)	0.4355 (4)	0.0146 (4)	0.0548 (11)	
H2A	0.2642	0.5277	0.0288	0.066*	
C10	0.5934 (5)	0.9530 (4)	0.2983 (4)	0.0592 (12)	
H10	0.6457	1.0527	0.3111	0.071*	
N2	0.7780 (4)	0.3664 (4)	0.4182 (3)	0.0764 (11)	
H2B	0.7646	0.4074	0.4886	0.092*	
H2C	0.8497	0.3332	0.4116	0.092*	
C9	0.4574 (5)	0.9015 (4)	0.3424 (4)	0.0599 (11)	
H9	0.4176	0.9657	0.3844	0.072*	
C8	0.3784 (5)	0.7539 (4)	0.3248 (3)	0.0534 (10)	
H8	0.2857	0.7189	0.3553	0.064*	
C15	0.6044 (4)	0.2679 (4)	0.0989 (3)	0.056	
H15	0.6170	0.2208	0.0228	0.068*	
C3	0.0530 (5)	0.3608 (5)	−0.0918 (4)	0.0677 (13)	
H3	0.0555	0.4027	−0.1486	0.081*	
N3	−0.2044 (5)	0.0239 (4)	−0.0519 (4)	0.106	
H3A	−0.2077	−0.0164	−0.0005	0.127*	
H3B	−0.2811	−0.0214	−0.1178	0.127*	
C4	−0.0726 (5)	0.2259 (5)	−0.1117 (4)	0.0687 (13)	
H4	−0.1568	0.1768	−0.1822	0.082*	
C20	0.0756 (6)	0.2038 (6)	0.4755 (5)	0.0963 (19)	
H20A	0.1265	0.2969	0.5458	0.116*	
H20B	−0.0150	0.1328	0.4941	0.116*	
C19	0.1946 (8)	0.1515 (7)	0.4477 (6)	0.147 (3)	
H19A	0.1414	0.0555	0.3825	0.221*	
H19B	0.2426	0.1447	0.5180	0.221*	
H19C	0.2800	0.2189	0.4239	0.221*	

### Atomic displacement parameters (Å^2^)

**Table:**

	*U*^11^	*U*^22^	*U*^33^	*U*^12^	*U*^13^	*U*^23^
P1	0.041	0.039	0.040	0.017	0.012	0.016
O1	0.051	0.050	0.044	0.021	0.021	0.021
O2	0.0604 (18)	0.062 (2)	0.081 (2)	0.0167 (15)	0.0085 (17)	0.0396 (17)
C12	0.050 (2)	0.036 (2)	0.042 (2)	0.0173 (19)	0.0080 (19)	0.0098 (18)
C7	0.043 (2)	0.037 (2)	0.037 (2)	0.0159 (18)	0.0056 (18)	0.0149 (18)
C1	0.043 (2)	0.047 (2)	0.041 (2)	0.023 (2)	0.0107 (18)	0.0159 (19)
C13	0.041 (2)	0.030 (2)	0.039 (2)	0.0122 (17)	0.0108 (18)	0.0129 (17)
C17	0.054	0.046	0.040	0.025	0.012	0.016
C11	0.049	0.045	0.042	0.012	0.007	0.019
C18	0.049	0.039	0.042	0.023	0.013	0.011
N1	0.082	0.056	0.078	0.021	0.031	0.024
C14	0.061	0.057	0.040	0.038	0.016	0.021
C16	0.056	0.055	0.056	0.034	0.021	0.024
C6	0.041 (2)	0.048 (2)	0.053 (3)	0.018 (2)	0.012 (2)	0.019 (2)
C5	0.038	0.042	0.067	0.013	0.007	0.001
C2	0.051 (2)	0.058 (3)	0.052 (3)	0.021 (2)	0.007 (2)	0.022 (2)
C10	0.071 (3)	0.038 (2)	0.056 (3)	0.017 (2)	−0.001 (2)	0.017 (2)
N2	0.096 (3)	0.104 (3)	0.046 (2)	0.076 (2)	0.004 (2)	0.014 (2)
C9	0.068 (3)	0.049 (3)	0.056 (3)	0.030 (2)	0.004 (2)	0.011 (2)
C8	0.056 (2)	0.048 (3)	0.052 (3)	0.024 (2)	0.007 (2)	0.016 (2)
C15	0.073	0.064	0.042	0.039	0.020	0.021
C3	0.070 (3)	0.071 (3)	0.058 (3)	0.033 (3)	0.006 (3)	0.023 (3)
N3	0.089	0.071	0.110	0.009	−0.009	0.020
C4	0.062 (3)	0.076 (3)	0.057 (3)	0.035 (3)	−0.004 (2)	0.013 (3)
C20	0.072 (3)	0.072 (4)	0.140 (6)	0.024 (3)	0.002 (4)	0.054 (4)
C19	0.158 (6)	0.134 (6)	0.132 (6)	0.039 (5)	−0.018 (5)	0.077 (5)

### Geometric parameters (Å, °)

**Table:**

P1—O1	1.500 (2)	C6—C5	1.385 (5)
P1—C13	1.794 (3)	C6—H6	0.9300
P1—C7	1.799 (3)	C5—N3	1.362 (5)
P1—C1	1.799 (4)	C5—C4	1.393 (5)
O2—C20	1.441 (5)	C2—C3	1.394 (5)
O2—H2	0.8200	C2—H2A	0.9300
C12—C7	1.381 (5)	C10—C9	1.361 (5)
C12—C11	1.398 (5)	C10—H10	0.9300
C12—H12	0.9300	N2—H2B	0.8600
C7—C8	1.388 (5)	N2—H2C	0.8600
C1—C2	1.381 (5)	C9—C8	1.383 (5)
C1—C6	1.399 (5)	C9—H9	0.9300
C13—C14	1.376 (5)	C8—H8	0.9300
C13—C18	1.380 (4)	C15—H15	0.9300
C17—N2	1.370 (4)	C3—C4	1.361 (5)
C17—C18	1.390 (4)	C3—H3	0.9300
C17—C16	1.396 (5)	N3—H3A	0.8600
C11—N1	1.362 (4)	N3—H3B	0.8600
C11—C10	1.387 (5)	C4—H4	0.9300
C18—H18	0.9300	C20—C19	1.424 (8)
N1—H1A	0.8600	C20—H20A	0.9700
N1—H1B	0.8600	C20—H20B	0.9700
C14—C15	1.377 (5)	C19—H19A	0.9600
C14—H14	0.9300	C19—H19B	0.9600
C16—C15	1.372 (5)	C19—H19C	0.9600
C16—H16	0.9300		
			
O1—P1—C13	112.04 (15)	N3—C5—C4	120.3 (4)
O1—P1—C7	110.89 (16)	C6—C5—C4	119.1 (4)
C13—P1—C7	107.89 (16)	C1—C2—C3	120.6 (4)
O1—P1—C1	112.16 (15)	C1—C2—H2A	119.7
C13—P1—C1	106.99 (16)	C3—C2—H2A	119.7
C7—P1—C1	106.60 (16)	C9—C10—C11	121.7 (4)
C20—O2—H2	109.5	C9—C10—H10	119.2
C7—C12—C11	121.1 (4)	C11—C10—H10	119.2
C7—C12—H12	119.5	C17—N2—H2B	120.0
C11—C12—H12	119.5	C17—N2—H2C	120.0
C12—C7—C8	119.3 (3)	H2B—N2—H2C	120.0
C12—C7—P1	122.6 (3)	C10—C9—C8	120.1 (4)
C8—C7—P1	118.1 (3)	C10—C9—H9	120.0
C2—C1—C6	119.5 (3)	C8—C9—H9	120.0
C2—C1—P1	122.8 (3)	C9—C8—C7	120.0 (4)
C6—C1—P1	117.7 (3)	C9—C8—H8	120.0
C14—C13—C18	120.2 (3)	C7—C8—H8	120.0
C14—C13—P1	122.0 (3)	C16—C15—C14	120.6 (4)
C18—C13—P1	117.8 (3)	C16—C15—H15	119.7
N2—C17—C18	121.5 (3)	C14—C15—H15	119.7
N2—C17—C16	121.0 (3)	C4—C3—C2	119.3 (4)
C18—C17—C16	117.5 (3)	C4—C3—H3	120.4
N1—C11—C10	120.0 (4)	C2—C3—H3	120.4
N1—C11—C12	122.2 (4)	C5—N3—H3A	120.0
C10—C11—C12	117.8 (4)	C5—N3—H3B	120.0
C13—C18—C17	121.3 (3)	H3A—N3—H3B	120.0
C13—C18—H18	119.4	C3—C4—C5	121.6 (4)
C17—C18—H18	119.4	C3—C4—H4	119.2
C11—N1—H1A	120.0	C5—C4—H4	119.2
C11—N1—H1B	120.0	C19—C20—O2	109.0 (5)
H1A—N1—H1B	120.0	C19—C20—H20A	109.9
C13—C14—C15	119.4 (3)	O2—C20—H20A	109.9
C13—C14—H14	120.3	C19—C20—H20B	109.9
C15—C14—H14	120.3	O2—C20—H20B	109.9
C15—C16—C17	121.0 (4)	H20A—C20—H20B	108.3
C15—C16—H16	119.5	C20—C19—H19A	109.5
C17—C16—H16	119.5	C20—C19—H19B	109.5
C5—C6—C1	120.0 (4)	H19A—C19—H19B	109.5
C5—C6—H6	120.0	C20—C19—H19C	109.5
C1—C6—H6	120.0	H19A—C19—H19C	109.5
N3—C5—C6	120.6 (4)	H19B—C19—H19C	109.5
			
C11—C12—C7—C8	0.0 (5)	N2—C17—C18—C13	−178.7 (3)
C11—C12—C7—P1	179.4 (3)	C16—C17—C18—C13	−0.6 (5)
O1—P1—C7—C12	148.9 (3)	C18—C13—C14—C15	−0.6 (5)
C13—P1—C7—C12	25.9 (3)	P1—C13—C14—C15	178.5 (3)
C1—P1—C7—C12	−88.7 (3)	N2—C17—C16—C15	178.3 (3)
O1—P1—C7—C8	−31.7 (3)	C18—C17—C16—C15	0.2 (5)
C13—P1—C7—C8	−154.7 (3)	C2—C1—C6—C5	−1.4 (5)
C1—P1—C7—C8	90.7 (3)	P1—C1—C6—C5	178.1 (3)
O1—P1—C1—C2	137.1 (3)	C1—C6—C5—N3	−179.3 (4)
C13—P1—C1—C2	−99.7 (3)	C1—C6—C5—C4	0.3 (6)
C7—P1—C1—C2	15.5 (4)	C6—C1—C2—C3	1.4 (6)
O1—P1—C1—C6	−42.5 (3)	P1—C1—C2—C3	−178.1 (3)
C13—P1—C1—C6	80.8 (3)	N1—C11—C10—C9	179.1 (3)
C7—P1—C1—C6	−164.0 (3)	C12—C11—C10—C9	−0.3 (6)
O1—P1—C13—C14	145.0 (3)	C11—C10—C9—C8	0.4 (6)
C7—P1—C13—C14	−92.6 (3)	C10—C9—C8—C7	−0.2 (6)
C1—P1—C13—C14	21.7 (3)	C12—C7—C8—C9	0.0 (5)
O1—P1—C13—C18	−35.8 (3)	P1—C7—C8—C9	−179.4 (3)
C7—P1—C13—C18	86.5 (3)	C17—C16—C15—C14	−0.1 (6)
C1—P1—C13—C18	−159.2 (3)	C13—C14—C15—C16	0.3 (5)
C7—C12—C11—N1	−179.3 (3)	C1—C2—C3—C4	−0.1 (6)
C7—C12—C11—C10	0.1 (5)	C2—C3—C4—C5	−1.0 (6)
C14—C13—C18—C17	0.8 (5)	N3—C5—C4—C3	−179.5 (4)
P1—C13—C18—C17	−178.4 (3)	C6—C5—C4—C3	1.0 (6)

### Hydrogen-bond geometry (Å, °)

**Table:**

*D*—H···*A*	*D*—H	H···*A*	*D*···*A*	*D*—H···*A*
N3—H3A···N1^i^	0.86	2.62	3.469 (6)	168
O2—H2···O1	0.82	1.85	2.672 (3)	178
N2—H2B···O1^ii^	0.86	2.14	2.987 (4)	168
N2—H2C···O2^iii^	0.86	2.23	3.089 (5)	173

Symmetry codes: (i) *x*−1, *y*−1, *z*; (ii) −*x*+1, −*y*+1, −*z*+1; (iii) *x*+1, *y*, *z*.
